# Health and Demographic Surveillance Systems Within the Child Health and Mortality Prevention Surveillance Network

**DOI:** 10.1093/cid/ciz609

**Published:** 2019-10-09

**Authors:** Solveig A Cunningham, Nida I Shaikh, Ariel Nhacolo, Pratima L Raghunathan, Karen Kotloff, Abu Mohd Naser, Melkamu M Mengesha, Sunday A Adedini, Thomas Misore, Uma U Onuwchekwa, Mary Claire Worrell, Shams El Arifeen, Nega Assefa, Atique I Chowdhury, Reinhard Kaiser, Shabir A Madhi, Ashka Mehta, David Obor, Charfudin Sacoor, Samba O Sow, Milagritos D Tapia, Amanda L Wilkinson, Robert F Breiman, Child Health and Mortality Prevention Surveillance (CHAMPS) Methods Writing Consortium, Janet Agaya, George Aol, Stephen Liech, Leonard Oyuga, Victor Akelo, Beth A Tippett Barr, Emily Zielinski-Gutierrez, Sanwarul Bari, Qazi Sadequr Rahman, Md. Mamunur Rashid, Tanvir Hossain, Quique Bassat, Quique Bassat, Paulo Filimone, Aura Hunguana, Edgar Jamisse, Teodimiro Matsena, Inacio Mandomando, Arlindo Malheia, Inacio Mandomando, Dickens Onyango, Matshidiso Sello, Dineo Thaele, Amara Jambai

**Affiliations:** 1 Emory Global Health Institute, Emory University, Atlanta, Georgia, USA; 2 Centro de Investigação em Saúde de Manhiça (CISM), Maputo, Mozambique; 3 Center for Global Health, Centers for Disease Control and Prevention, Atlanta, Georgia, USA; 4 Department of Pediatrics, Center for Vaccine Development and Global Health, University of Maryland School of Medicine, Baltimore, Maryland, USA; 5 College of Health and Medical Sciences, Haramaya University, Harar, Ethiopia; 6 Medical Research Council, Respiratory and Meningeal Pathogen Research Unit, University of the Witwatersrand, Faculty of Health Sciences, Johannesburg, South Africa; 7 Demography and Population Studies Program, Schools of Public Health and Social Sciences, University of the Witwatersrand, Johannesburg, South Africa; 8 Kenya Medical Research Institute, Kisumu, Kenya; 9 Centre pour le Développement des Vaccins (CVD-Mali), Ministère de la Santé, Bamako, Mali; 10 Maternal and Child Health Division, icddr,b, Dhaka, Bangladesh; 11 US Centers for Disease Control and Prevention–Sierra Leone, Freetown, Sierra Leone; 12 Department of Science and Technology/National Research Foundation, Vaccine Preventable Diseases, University of the Witwatersrand, Faculty of Health Sciences, Johannesburg, South Africa; 13 Kenya Medical Research Institute, Kisumu; 14 US Centers for Disease Control and Prevention–Kenya, Nairobi Kenya; 15 PEI, Infectious Disease Division, icddr,b, Dhaka, Bangladesh; 16 ISGlobal, Hospital Clínic, Universitat de Barcelona/ICREA/Pediatric Infectious Diseases Unit, Pediatrics Department, Hospital Sant Joan de Déu, Universitat de Barcelona, Spain; 17 Consorcio de Investigacion Biomedica en Red de Epidemiologia y Salud, Spain; 18 Centro de Investigação em Saúde de Manhiça [CISM], Maputo, Mozambique; 19 Instituto Nacional de Saude, Ministerio de Saude, Maputo, Mozambique; 20 Kisumu County Department of Health, Kisumu, Kenya; 21 Respiratory and Meningeal Pathogens Research Unit, University of the Witwatersrand, Johannesburg, South Africa; 22 Ministry of Health and Sanitation, Sierra Leone

**Keywords:** demographic surveillance, child death, population health, data methodology

## Abstract

Health and demographic surveillance systems (HDSSs) provide a foundation for characterizing and defining priorities and strategies for improving population health. The Child Health and Mortality Prevention Surveillance (CHAMPS) project aims to inform policy to prevent child deaths through generating causes of death from surveillance data combined with innovative diagnostic and laboratory methods. Six of the 7 sites that constitute the CHAMPS network have active HDSSs: Mozambique, Mali, Ethiopia, Kenya, Bangladesh, and South Africa; the seventh, in Sierra Leone, is in the early planning stages. This article describes the network of CHAMPS HDSSs and their role in the CHAMPS project. To generate actionable health and demographic data to prevent child deaths, the network depends on reliable demographic surveillance, and the HDSSs play this crucial role.

Demographic surveillance systems (DSSs) are commonly used to monitor populations and their health over time within geographically defined demographic surveillance areas (DSAs). DSSs are longitudinal data collection platforms that track births, deaths, migrations, and socioeconomic and health circumstances over time in places where vital statistics are not reliably collected. When disease surveillance, either passive, through hospital-based surveillance, or active, through community surveillance, is attached to the DSS, the whole platform is called a health and demographic surveillance system (HDSS).

A key objective of the Child Health and Mortality Prevention Surveillance project is to define population-based rates for definitive causes of death through diagnostic and laboratory methods nested within population surveillance [1]. The CHAMPS HDSSs provide this platform, including through demographic data for estimating population-based mortality rates and contextual information for understanding factors associated with the deaths of children <5 years of age. The CHAMPS investments since 2016 have led to the modification and expansion of HDSSs that already existed in Mozambique, Mali, Ethiopia, and Kenya and to the establishment of new sites in South Africa and Bangladesh and, in the future, in Sierra Leone.

## HEALTH AND DEMOGRAPHIC SURVEILLANCE SYSTEMS IN CHAMPS

HDSSs monitor population dynamics and population health, including demographic events, social and economic conditions, health-seeking behaviors, pregnancies, and disease outbreaks. HDSSs also provide demographically characterized sampling frames from which representative samples can be selected; this is a platform for conducting surveys, demonstration projects, and effectiveness studies, implementing and evaluating interventions, and carrying out investigational trials for new products with potential public health value [2–4]. Longitudinal population monitoring is accomplished through enumeration of all residents of a geographically defined area and routine visits at least 2 times per year to all homesteads to collect data on all births and other pregnancy outcomes, deaths, migrations into, out of, and within the DSA, and additional information relevant to health [5–8]. HDSSs also obtain cause of death information through verbal autopsies; this involves interviews with family members of deceased individuals about the symptoms and circumstances surrounding the death, which are analyzed through computer-based algorithms and physician adjudication panels [5, 9]. Many HDSSs also geocode homesteads, infrastructure, and community landmarks, such as clinics and hospitals.

CHAMPS uses age- and sex-specific population counts and person-years lived at each site to calculate rates, including infant mortality rates, under-5 mortality rates, and stillbirth rates. This information is also used to assess the proportion of child deaths that undergo minimally invasive tissue sampling (MITS) procedures and the proportion of deaths for which the cause was identified through verbal autopsies or clinical records [1, 10]. The information collected by the HDSSs on households’ demographic and socioeconomic characteristics, access to healthcare, and health facilities in the DSA provides contextual information for child mortality, helping the CHAMPS project to identify factors contributing to mortality and opportunities for interventions.

Given that HDSSs have long been in operation in several CHAMPS sites, there is some variability in instruments and methodology used. At the same time, they share similarities in core conceptual, methodological, and community engagement procedures. The CHAMPS network balances the need for standardized, comparable data collection with recognition that each site has different circumstances and needs to contribute to local priorities, circumstances, and research and policy interests. Sites maintain well-trained and supervised staff, use documented field methods, implement data monitoring and data management procedures, and engage in data analysis and dissemination. They emphasize ethical, respectful data collection, reporting methods and community engagement.

Table 1 shows the data elements used in CHAMPS surveillance. For CHAMPS, the priority HDSS data are demographic characteristics, specifically, age- and sex- specific population and demographic events that change the population: births, deaths, and in- and out-migrations. A secondary priority is contextual information about children’s circumstances that may be related to their health and survival. This includes characteristics of mothers, households and homesteads, and healthcare utilization. Thus, many HDSSs collect information on household members’ education and marital status; mothers’ pregnancy history, antenatal care, and delivery; children’s immunization history; household assets, water and sanitation practices, and malaria control measures; and homestead building materials of dwellings and number of rooms. As HDSSs become well established and explore more diverse approaches to understanding child health and mortality, additional relevant indicators are considered, such as anthropometric measurements, anemia assessment, HIV testing, and 24-hour dietary recall.

**Table 1.** Data Elements in Child Health and Mortality Prevention Surveillance

Data Elements	Description
Core indicators	The minimum data elements essential for calculating mortality rates: age- and sex-specific population size, age- and sex-specific numbers of deaths, sex-specific number of births; number of in- and out-migrations
Household and individual indicators	These data elements are important for contextualizing CHAMPS results, and provide opportunities for examining the relationships between environmental factors and child mortality
	• Households: water and sanitation, cooking fuel, socioeconomic status, distance and access to healthcare, household composition
	• Mothers/adults: age, education, reproductive history
	• Children: antenatal, delivery, and postnatal care, breastfeeding, and immunizations
Biomarkers and nutrition indicators	These are elements considered for future expansion of data collection, as they can provide new directions for understanding maternal and child health. • Biomarkers: height, weight, and other anthropometric measurements, hemoglobin and anemia assessment, HIV testing
	• 24-hour dietary recall or food frequency questionnaires adapted to the local context

Abbreviations: CHAMPS, Child Health and Mortality Prevention Surveillance; HIV, human immunodeficiency virus.

CHAMPS maintains a Program Office, which monitors and evaluates procedures and data quality at each HDSS, works with HDSSs to assess their capacity to collect quality data, and identifies needs and priorities for capacity improvements. Each HDSS reports aggregate data after each data round and also reports final, cleaned data at the end of each year. At the first stage, these are aggregate data on demographic events (eg, births, deaths) and population counts for each sex and age group. On-site assessments and technical assistance are provided when a site or the Program Office identifies a need. When needed, CHAMPS partners with local and regional demographic surveillance experts to build capacity. Knowledge-sharing across CHAMPS HDSSs is supported through regular teleconferences, site exchange visits, and in-person meetings.

## HDSS SITE PROFILES

Characteristics of the CHAMPS HDSS network are presented below and summarized in Table 2. Table 3 provides an overview of the data collected currently across the HDSSs.

**Table 2.** Characteristics of Child Health and Mortality Prevention Surveillance Health and Demographic Surveillance Systems

Characteristic	Mozambique	Mali	Ethiopia		Kenya		Bangladesh	South Africa
Year HDSS established	1996	2006	2007	2012	2007	2016	2017	2017
Site location	Manhiça	Djicoroni Para & Banconi, Bamako	Kersa	Harar	Siaya, Karemo	Manyatta	Baliakandi	Soweto & Thembelihle
Site setting	Rural	Urban	Rural	Urban	Rural	Urban	Rural	Urban
Area covered, km^2^	2380	11.15	353	25.4	200	5	242.5	36.7
Size of population covered 2017–2018	186 000	227 219	131 431	60 044	93 000	77 000	216 362	123 225
Population density, persons/km^2^	78	20 378	371	2362	465	14 400	892	6034
Annual data collection rounds	2	2	2	2	2	2	Current: 3	Current: 2
							Planned: 6	Planned: 3
Staff	71	143	80	40	31	25	27	40
Fieldworkers	53	137	62	25	…	20	19	35
Data entry and management	15	5	12	9	…	5	7	2
Scientific staff	3	1	6	6	…	…	1	3

An HDSS is being planned in Sierra Leone in Bombali Shebora and Bombali Siari covering a population of 162 000.

Abbreviation: HDSS, health and demographic surveillance system.

**Table 3.** Overview of Typical Data Elements Collected by Child Health and Mortality Prevention Surveillance Health and Demographic Surveillance Systems in 2017–2019

Characteristic	Mozambique	Mali	Ethiopia	Kenya	Bangladesh	South Africa
Household demographic information						
Age and sex of members	√	√	√	√	√	√
Births and deaths	√	√	√	√	√	√
Pregnancy status and history	√	√	√	√	√	√
In- and out-migration	√	√	√	√	√	√
Characteristics of household members						
Marital status	√	…	√	√	√	√
Religion	√	…	√	√	√	√
Ethnicity languages spoken	√	…	√	√	…	√
Education	√	…	√	√	√	√
Occupation	√	…	√	√	√	√
Household socioeconomic status						
Income	…	…	√	…	…	…
Expenditure	√	…	…	…	…	…
Ownerships of homestead or land	…	…	√	√	√	√
Livestock	√	…	√	√	√	…
Household assets	√	…	√	√	√	√
Homestead characteristics						
Homestead structure	…	…	…	…	…	…
Wall	√	…	√	…	√	√
Floor	√	…	√	√	√	√
Roof	√	…	√	√	√	√
Windows	√	…	…	…	…	…
Drinking water practices	√	…	√	√	√	√
Sanitation practices	√	…	√	√	√	√
Cooking fuel and stoves	√	…	√	√	…	√
Health of household members						
Health conditions	√	…	√	√	…	√
Symptoms of children’s malnutrition	…	…	√	…	…	…
Disease prevention						
Immunization of under-5 children	√	√	√	√	…	√
Mosquito bite prevention	√	…	…	…	…	…
Healthcare utilization	…	…	√	√	…	…

“√” indicates that the HDSS data instruments include 1 or more questions on the topic. There are differences across HDSS sites in question wording and response options. Some HDSSs will begin collecting additional variables in 2020.

Abbreviation: HDSS, health and demographic surveillance system.

## Manhiça HDSS, Mozambique

The longest-running HDSS participating in the CHAMPS network was established in 1996 by the Manhiça Health Research Center (CISM). It is one of the founding member of the International Network for the Demographic Evaluation of Populations and their Health (INDEPTH) Network of HDSS. It is located in Manhiça district, about 85 km north of Maputo City, the capital of Mozambique. It initially covered an area of 100 km^2^ with 32 000 people [2, 11]. The HDSS, with a staff of 71 personnel, was expanded in 2002 to an area of 450 km [2], in 2005 to an area of 500 km^2^, and in 2014 to cover the entire district of Manhiça (2380 km^2^ and 186 000 people). Initially, three rounds of data collection were conducted each year until 2001 when they were reduced to two, and to one per year during the period 2013 to 2016; they increased again to two rounds each year in 2017 afterwards. Each round includes modules to update the household and individual data and, where applicable, modules for migration, pregnancies, pregnancy outcomes and fertility histories. Other modules collect data on water and sanitation, household assets, malaria prevention information, and immunization of children <5 years of age. All deaths are documented, including date, place, and details about cause of death using verbal autopsy methods. A link between demographic and ongoing childhood morbidity surveillance is made through individual demographic identification cards printed from the HDSS databases and distributed to the households for each child <15 years old. Key informants selected in each community provide alerts about deaths, births, marriages, and new households to HDSS supervisors who visit them every week. Some modifications in procedures were made to adapt the HDSS for CHAMPS. Verbal autopsy forms have been upgraded to the CHAMPS/World Health Organization (WHO) 2016 version in 2017 [12]. To meet CHAMPS needs, the period to contact families for verbal autopsies interview has been shortened from 3 months to 2–4 weeks after death. In addition, the site implemented a call center, which the key informants can contact to report demographic events in the community immediately 24 hours a day, 7 days a week. More information about the Manhiça HDSS and its findings has been published elsewhere [11, 13].

## Bamako Site, Mali

An HDSS was established in 2006 in 2 low-income urban communities of Djicoroni Para and Banconi, in Bamako, the capital of Mali. The HDSS, with a staff of 143 personnel, covers an area of 11.15 km^2^ with 227 219 people. An average of two rounds of demographic data have been conducted annually. The relationship with CHAMPS began in 2017. A network of informants provides alerts about births, deaths, and pregnancies in the community. The HDSS has also served as a platform for multiple studies, including serologic surveys, to assess, the impact of the introduction of *Haemophilus influenzae* type b vaccine [14] and the prevalence of echocardiogram-diagnosed rheumatic heart disease, healthcare utilization surveys [15], and randomized selection for case-control studies for the Global Enteric Multicenter Study (GEMS) [16] and the Vaccine Impact on Diarrhea in Africa (VIDA) study. In 2017, the verbal autopsy forms were updated to collect information on stillbirths and to correspond with the CHAMPS/WHO 2016 version. The HDSS is exploring methods for early pregnancy detection through community-based last menstrual period tracking with the assistance of a network of midwives.

## Kersa and Harar Sites, Ethiopia

There are 2 HDSSs associated with the CHAMPS site in Ethiopia: 1 in the rural region of Kersa, established in 2007, and the other in the urban region of the Harari Regional State, established in 2012. At onset, the system in Kersa included 12 subdistricts (*kebeles*) with a total population of 52 000 and in Harar included 6 kebeles with 34 000 inhabitants. In 2014, other kebeles were added, doubling the catchment area of each site. Currently, Kersa HDSS has 24 kebeles and covers 353 km^2^ and a population of 131 431. Harar HDSS has 12 kebeles, covering 25.4 km^2^ and a population of 60 044. There are 80 and 40 HDSS staff members at Kersa and Harar, respectively. Two data rounds are conducted per year, during which demographic and health-related information is collected, including immunization of children, morbidity, family planning, and verbal autopsies [17, 18].

## Siaya-Karemo and Manyatta Sites, Kenya

There are 2 HDSSs associated with the CHAMPS project in Kenya. A rural HDSS was set up in the districts of Asembo and Gem, Siaya County, in 2001 and 2002, respectively. In 2007, surveillance was expanded to the Karemo Division of Siaya County. The Karemo surveillance area is part of the CHAMPS network. As of 2014, Karemo covers a population of 93 000 in approximately 200 km^2^. In addition, an urban HDSS was established in Manyatta in 2016 for CHAMPS, adapting the procedures from the rural sites to an urban population. As of 2018, it covered 77 000 people in 5 km^2^. The sites conduct 2 data rounds annually. There are 31 and 25 staff members at the Siaya and Manyatta HDSS locations, respectively. The verbal autopsy procedures were updated to collect information on stillbirths and to correspond with the CHAMPS/WHO 2016 version. More information about the HDSS and its findings has been published elsewhere [6, 19].

## Baliakandi Site, Bangladesh

This HDSS was established by icddr,b for CHAMPS in rural Rajbari district in March, 2017. The HDSS covers the entire *upazila* (subdistrict) of Baliakandi, with an area of 242.5 km^2^ and population of 216 362. Currently, it conducts 6 data rounds per year to accommodate a detailed pregnancy surveillance component. The HDSS collects information on demographic events; household assets; education and occupation of household members; pregnancy history of all married women of reproductive age; and geospatial information on homesteads, health facilities, and landmarks. There are 27 staff members.

## Soweto and Thembelihle Site, South Africa

An HDSS was initiated for CHAMPS in Soweto and Thembelihle in 2017–2018. Soweto is an urban township with around 1.3 million individuals covering an area of 200 km^2^ in about 100 subplaces; Thembelihle Local Municipality and its adjoining areas are informal urban settlements with a population of >20 000. The HDSS catchment areas cover 8 mostly noncontiguous subplaces of low socioeconomic status, with an area of 17.7 km [2] in Soweto; Thembelihle and its surrounding informal settlements has an area of 19.0 km [2]. The population under surveillance in 2018 was 123 225 individuals in 35 302 households. The HDSS is conducting 2 rounds of household visits per year with a staff of 40 members, collecting demographic and socioeconomic information, geographic indicators, pregnancy history, pregnancy outcomes, child health, and migration.

## Bombali Shebora and Bombali Siari, Sierra Leone

The CHAMPS surveillance area in Sierra Leone does not have an HDSS yet, but is in the early stages of planning for one. The HDSS is expected to comprise 2 chiefdoms, with a population of 161 000 in an area of 281.7 km^2^: Bombali Shebora, which includes Makeni City, and Bombali Siari. CHAMPS is working with local partners to assess local capacity for establishing and maintaining an HDSS and engaging government and other possible national and international stakeholders.

## DISCUSSION

The CHAMPS network of HDSSs is a collaborative undertaking of independent, yet connected research groups. They share the common goal to collect high-quality data that can be used to characterize and prevent child mortality; each also has methods, priorities, collaborations, and challenges that are specific to the community and country within which it operates. As the network of HDSSs develops, several opportunities and challenges emerge.

### Challenges

HDSS capacity at some sites was established prior to engagement with CHAMPS, while at other sites an HDSS is being newly established through CHAMPS financial and technical support. As a result, instruments, field methods, and data processes differ across sites and are not standardized. CHAMPS Program Office experts work with scientific staff and leadership at each site to systematically assess needs pertaining to data collection protocols, fieldwork procedures, data entry and management, and technology and software. They ensure that the data meet CHAMPS requirements, and that site-to-site differences are understood and considered during calculations of the indices and interpretation of results.

CHAMPS requires extensive community engagement and trust. The constant presence of the HDSS in communities through visits to all households every few months for data rounds can be a platform for CHAMPS to build the rapport and trust in communities to work around the sensitive subject of child mortality.

Some sites are conducting HDSS activities in particularly challenging settings. For example, several sites cover urban areas, where individuals and households are frequently moving and changing circumstances, so they are difficult to track over time. A few HDSSs have noncontiguous DSAs, which makes it difficult to ensure that the catchment areas are well demarcated and to track the population without double-counting movers; it also makes travel to households longer for data collection and supervision.

For CHAMPS sites that are newly developing, there are challenges in creating standard operating procedures, establishing both cause of death–related activities and HDSS activities simultaneously, and training new staff and creating collaborative teams. In some countries, there is limited local availability of demographic expertise; in some communities, hiring and specialized training is needed to develop a team of qualified staff for data collection and data management.

Tracking large populations over time is intensive and expensive, as it requires sufficient qualified locally based staff with long-term commitment to the study area. Developing and maintaining a quality HDSS is also scientifically challenging, requiring expertise in demography and epidemiology, survey and fieldwork methodology, data processing, and analysis and scientific writing. Sustained financial investments are required to support the staff and infrastructure necessary to visit tens or hundreds of thousands of individuals multiple times per year and collect, manage, and analyze the resulting data. However, funds are often difficult to attract and maintain for ongoing surveillance. Yet, in low-resource settings with limited or no vital registration, HDSSs, like the ones operating within the CHAMPS network, are the only source of much-needed information on population health.

### Lessons Learned

The CHAMPS network provides opportunities for HDSSs to share and exchange expertise, survey instruments and indicators, and methodologies for dealing with both routine and unusual research circumstances. As with any scientific collaboration, each site must meet its multiple priorities, including research, programmatic, publication, and sustainability goals. One theme in the challenges and successes has been the necessity of community acceptance for successful implementation of surveillance. In-country expertise in demography and in-field methodology are assets, as is the establishment of standard operating procedures locally. The use of technology, such as tablets, for data collection can be useful, but is neither required for quality data, nor guarantees quality data.

### Future Directions

The CHAMPS project requires HDSS data rounds at least twice yearly, bridging a balance between resource constraints and the need to collect complete data on births, deaths, and population characteristics. Some HDSSs conduct 3 or more data rounds per year to achieve better tracking of pregnancies and migrations, which are needed for accurate estimates of population and mortality. A key value of HDSSs is the longitudinal tracking of populations, which makes it possible to document temporal trends. HDSSs contribute to filling knowledge gaps about child mortality by providing population-based enumeration of children and of deaths in a well-characterized population; these data are additionally valuable when linked with cause of death data [20]. Systematic surveillance of vital events in HDSSs helps MITS to be performed within the necessary short timeframe of 24 hours after death; it also provides data on mortality by age group and on the household and community contexts of child mortality.

As the network develops, a priority is to envision and specify scientifically the applicability of the mortality data to populations outside of the DSA. We are exploring statistical methods for how mortality rates, contextualized using HDSS data, can yield results applicable to broader geographic areas.

High-quality data on child health and mortality can provide actionable evidence for developing strategies and interventions to prevent child deaths. For this purpose, the CHAMPS network depends on HDSSs to conduct reliable demographic surveillance and provide data gathered and maintained using appropriate methods.

## Notes

***Child Health and Mortality Prevention Surveillance (CHAMPS) Methods Writing Consortium.*** Janet Agaya, George Aol, Stephen Liech, and Leonard Oyuga (Kenya Medical Research Institute, Kisumu); Victor Akelo, Beth A. Tippett Barr, and Emily Zielinski-Gutierrez (US Centers for Disease Control and Prevention–Kenya, Nairobi Kenya); Sanwarul Bari, Qazi Sadequr Rahman, Md. Mamunur Rashid, and Tanvir Hossain (PEI, Infectious Disease Division, icddr,b, Dhaka, Bangladesh; Quique Bassat (ISGlobal, Hospital Clínic, Universitat de Barcelona/ICREA/Pediatric Infectious Diseases Unit, Pediatrics Department, Hospital Sant Joan de Déu, Universitat de Barcelona, Spain; Consorcio de Investigacion Biomedica en Red de Epidemiologia y Salud, Spain); Quique Bassat, Paulo Filimone, Aura Hunguana, Edgar Jamisse, Teodimiro Matsena, Inacio Mandomando, and Arlindo Malheia (Centro de Investigação em Saúde de Manhiça [CISM], Maputo, Mozambique); Inacio Mandomando (Instituto Nacional de Saude, Ministerio de Saude, Maputo, Mozambique); Dickens Onyango (Kisumu County Department of Health, Kisumu, Kenya); Matshidiso Sello and Dineo Thaele (Respiratory and Meningeal Pathogens Research Unit, University of the Witwatersrand, Johannesburg, South Africa); and Amara Jambai (Ministry of Health and Sanitation, Sierra Leone).

***Acknowledgments.*** This work is possible due to the dedication of staff in each Health and Demographic Surveillance System, the contributions of stakeholders, and the support and enthusiasm of members of each community.

***Financial support.*** This work was supported by the Bill & Melinda Gates Foundation [OPP1126780]. The Ethiopia HDSS sites have been supported by the CDC and the Ethiopian Public Health Association, and are supported by Haramaya University. CISM receives core funding from the Mozambican government and Agencia Espanhola de Cooperacion Internacional para el Desarollo. Grants from Emory University also supported this work (to Milagritos Tapia, Karen Kotloff, and Ashka Mehta).

***Supplement sponsorship.*** This supplement is sponsored by the Emory Global Health Institute and the Bill & Melinda Gates Foundation.

***Potential conflicts of interest.*** S. A. M. has received grants from the Bill & Melinda Gates Foundation, Pfizer, Glaxo Smith Kline, Biovac, Novavax, and Sanofi Pasteur; and honoraria from The Bill & Melinda Gates Foundation. All other authors report no potential conflicts of interest. All authors have submitted the ICMJE Form for Disclosure of Potential Conflicts of Interest. Conflicts that the editors consider relevant to the content of the manuscript have been disclosed.
