# Erratum to: Health and Demographic Surveillance Systems Within the Child Health and Mortality Prevention Surveillance Network

**DOI:** 10.1093/cid/ciz1162

**Published:** 2019-12-27

An error appeared in the original publication of the following article [Cunningham SA, Shaikh NI, Nhacolo A, et al. Health and Demographic Surveillance Systems Within the Child Health and Mortality Prevention Surveillance Network. Clin Infect Dis 2019; https://doi.org/10.1093/cid/ciz609].

the Child Health and Mortality Prevention Surveillance (CHAMPS) Methods Consortium was inadvertently omitted from the author byline.

The article has been corrected, and the publisher regrets this error.

